# Cyclo­hexyl­methyl­ammonium *N*,*N*′-dicyclo­hexyl-*N*,*N*′-dimethyl-*N*′′-(2,2,2-trifluoro­acet­yl)phospho­nic triamide)

**DOI:** 10.1107/S1600536808040737

**Published:** 2008-12-10

**Authors:** Mohammad Yazdanbakhsh, Hossein Eshtiagh-Hosseini, Fahimeh Sabbaghi

**Affiliations:** a Department of Chemistry, Faculty of Sciences, Ferdowsi University of Mashhad, Mashhad 91779, Iran

## Abstract

In the salt, C_7_H_16_N^+^·C_16_H_28_F_3_N_3_O_2_P^−^, the P atom shows tetra­hedral coordination. Two ion pairs are linked by N—H⋯O hydrogen bonds across a center of inversion. The phosphoryl and carbonyl groups are staggered [O—P—N—C = 64.8 (3)°].

## Related literature

For alkali metal salts of dimethyl-*N*-trichlor­acetyl­amido­phosphate, see: Trush *et al.* (2005 ▶). For a related structure, see: Yazdanbakhsh & Sabbaghi (2007 ▶). For bond-length data, see: Corbridge (1995 ▶). For synthetic details, see: Shokol *et al.* (1969 ▶).
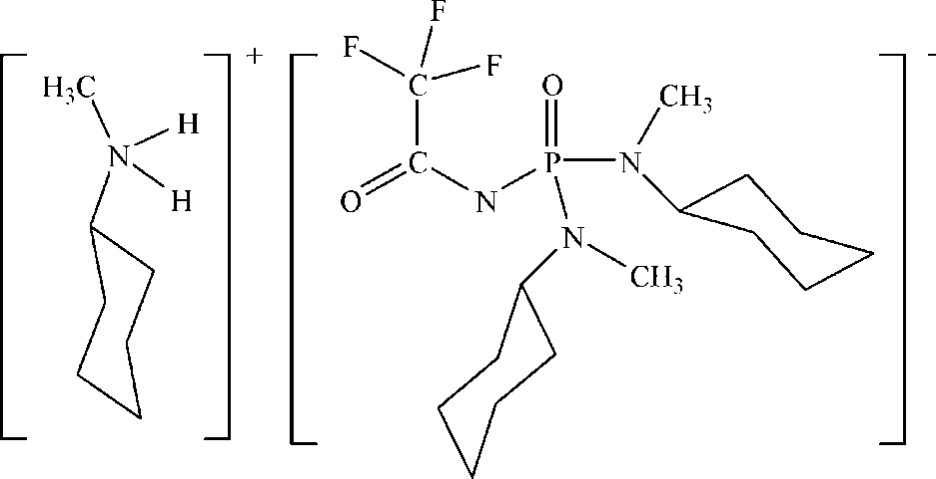


## Experimental

### 

#### Crystal data

C_7_H_16_N^+^·C_16_H_28_F_3_N_3_O_2_P^−^
*M* *_r_* = 496.59
Monoclinic, 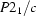

*a* = 9.183 (3) Å
*b* = 30.893 (7) Å
*c* = 9.241 (2) Å
β = 93.039 (7)°
*V* = 2617.9 (12) Å^3^
*Z* = 4
Mo *K*α radiation
μ = 0.15 mm^−1^
*T* = 120 (2) K
0.40 × 0.30 × 0.25 mm

#### Data collection

Bruker SMART 1000 CCD area-detector diffractometer
Absorption correction: multi-scan (*SADABS*; Sheldrick, 1996 ▶) *T*_min_ = 0.947, *T*_max_ = 0.969
23153 measured reflections
5148 independent reflections
2673 reflections with *I* > 2σ(*I*)
*R*_int_ = 0.064

#### Refinement

*R*[*F* ^2^ > 2σ(*F* ^2^)] = 0.060
*wR*(*F* ^2^) = 0.113
*S* = 1.08
5148 reflections
304 parameters
H-atom parameters constrained
Δρ_max_ = 0.33 e Å^−3^
Δρ_min_ = −0.32 e Å^−3^

### 

Data collection: *SMART* (Bruker, 1998 ▶); cell refinement: *SAINT-Plus* (Bruker, 1998 ▶); data reduction: *SAINT-Plus*; program(s) used to solve structure: *SHELXTL* (Sheldrick, 2008 ▶); program(s) used to refine structure: *SHELXTL*; molecular graphics: *DIAMOND* (Brandenburg, 2001 ▶); software used to prepare material for publication: *SHELXTL*.

## Supplementary Material

Crystal structure: contains datablocks I, global. DOI: 10.1107/S1600536808040737/ng2519sup1.cif

Structure factors: contains datablocks I. DOI: 10.1107/S1600536808040737/ng2519Isup2.hkl

Additional supplementary materials:  crystallographic information; 3D view; checkCIF report

## Figures and Tables

**Table 1:** Hydrogen-bond geometry (Å, °)

*D*—H⋯*A*	*D*—H	H⋯*A*	*D*⋯*A*	*D*—H⋯*A*
N4—H4*NA*⋯O1	0.95	1.84	2.771 (3)	167
N4—H4*NB*⋯O1^i^	0.95	1.87	2.804 (3)	168

Symmetry code: (i) 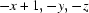.

**Fig. 1.**
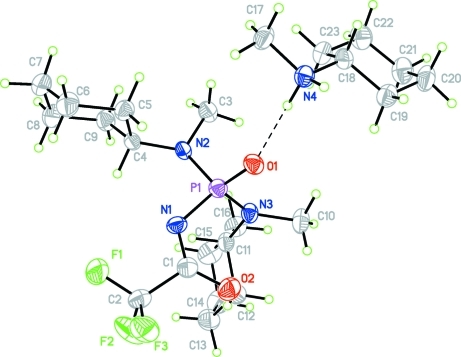
General view of [NH2CH3C6H11][CF3CONPO(NCH3C6H11)2] in representation of atoms via thermal ellipsoids at 50% probability level (all hydrogen atoms except H(4 N A) and H(4NB) are omitted for clarity).

**Fig. 2.**
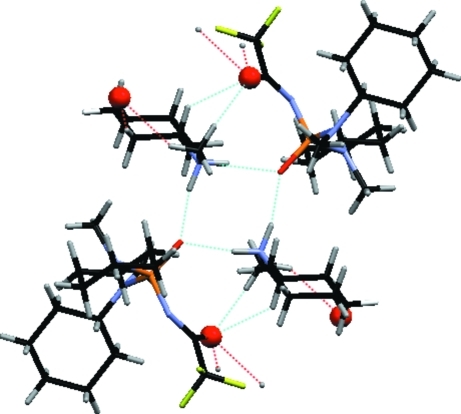
The fragment of crystal packing of [NH2CH3C6H11][CF3CONPO(NCH3C6H11)2] along the crystallographic plane ab (all hydrogen atoms except H(4 N A)and H(4NB) are omitted for clarity).

**Fig. 3.**
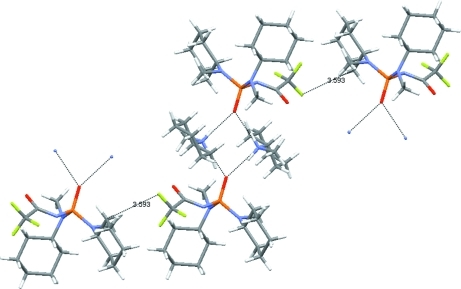
A view of C—H···O hydrogen bonds in [NH2CH3C6H11][CF3CONPO(NCH3C6H11)2].

**Fig. 4.**
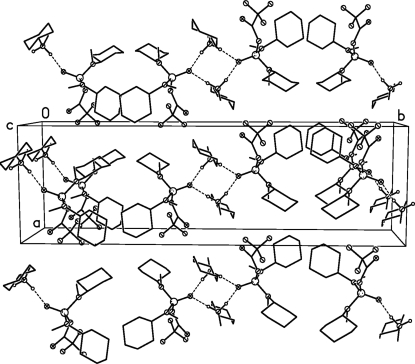
A view of C—H···F hydrogen bonds in [NH2CH3C6H11][CF3CONPO(NCH3C6H11)2].

## supplementary crystallographic information

### Comment

Alkali onic-salts (Na^+^, Rb^+^) of dimethyl-*N*-trichloracetylamidophosphate [HL] were synthesized from aqueous-alcoholic solutions (Trush *et al.*, 2005). Furthermore synthesis and investigation of onic-salts SbPh_4_^+^ allowed the determination of the preferable donor center of the deprotonated ligand (the oxygen atom of the phosphoryl group). Using non-coordinating ions PPh_4_^+^ permits to synthesize and characterize structurally of the "free" non-solvated [HL] anion. This information could be used for the molecular design of coordination systems based on carbacylamidophosphates. Here, we report on a new onic-salt (NH_2_CH_3_C_6_H_11_^+^) of [CF_3_CONPO(NCH_3_C_6_H_11_)_2_]^-^ obtained from a reaction between LiOH and ligand. Single crystal of the product [NH_2_CH_3_C_6_H_11_][CF_3_CONPO(NCH_3_C_6_H_11_)_2_] was obtained from a solution of CH_3_OH—H_2_O (3:1) after a slow evaporation at room temperature. The proton transfer compound contains *N*-methyl cyclohexyl ammonium cation and deprotonated *N*'-2,2,2,-tri-flouroacetyl bis *N*"-methyl cyclohexyl phosphortriamide (Fig. 1). The structure of the title compound is composed of centrosymmetric dimmers (of two bridged cations between two anions) forming by intermolecular N^+^—H···OP hydrogen bonds (N···O = 2.771 (3) Å & 2.804 (3) Å), Fig. 2. The phosphoryl and the carbonyl groups in the structure are not in *anti* position (O(1)—P(1)—N(1)—C(1) = 64.8 (3)°) against the previous reported carbacylamidophosphates (Yazdanbakhsh & Sabbaghi, 2007). The phosphorus atom has slightly distorted tetrahedral configuration. The bond angles around P(1) atom is in the range of 100.79 (12)°-115.26 (13)° that the highest and the lowest values were obtained for the angles *O*—*P*—*N*(1)_amide_ and *N*(2)_amine_—*P*—*N*(1)_amide_. The P(1)—N(1), P(1)—N(2) and P(1)—N(3) bond lengths are 1.629 (3) Å, 1.651 (2) Å and 1.643 (2) Å. They are significantly shorter than the typical P—N single bond length (1.77 Å) (Corbridge, 1995). Sum of the surrounding angles around N(2) and N(3) atoms are 353.5° and 356.0° that indicate some deviation from planarity. Furthermore the angle C(1)—N(1)—P(1) (123.4 (2)°) confirm the *sp*^2^ hybridization for the nitrogen atom. The PO bond length (1.511 (2) Å) is larger than the normal P?O bond length (1.45 Å). The CO group cooperates in weak C—H···O hydrogen bonds forming four hydrogen bonds with two neighboring cations (C(17)—H(17 C)···O(2)—C(1), C(17)···O(2) = 3.320 Å; C(18)—H(18 A)···O(2)—C(1), C(18)···O(2) = 3.214 Å; C(17)—H(17B)···O(2)—C(1), C(17)···O(2) = 3.365 Å; C(23)—H(23 A)···O(2)—C(1), C(23)···O(2) = 3.562 Å) (Fig. 3). Moreover, the C—H···F hydrogen bonds exist in the crystal network (C(5)—H(5 A)···F(3), C(5)···F(3) = 3.593 Å) (Fig. 4).

### Experimental

CF_3_C(O)N(H)P(O)Cl_2_ was prepared similar to the literature method (Shokol *et al.*, 1969) from the reaction of phosphorus pentachloride and 2,2,2-triflouoroacetamide in CCl_4_ and then the treatment of formic acid. Synthesis of CF_3_C(O)N(H)P(O)[N(CH_3_)(C_6_H_11_)]_2_ To a solution of (1.15 g, 5 mmol) triflouroacetyl phosphoramidic dichloride in CCl_4_ (20 ml), a solution of *N*-methylcyclohexylamine (2.26 g, 20 mmol) in CCl_4_ (10 ml) was added dropwise at 0°C. After 24 h stirring, the solvent was removed in vacuum and the solid product was washed with distilled water. The residue recrystallized in CH_3_CN. Anal. Calc. for C_16_H_29_F_3_N_3_O_2_P: C, 50.10; H, 7.56; N, 10.95. Found: C, 49.72; H, 7.84; N, 10.74%. ^31^P NMR ([D_6_]DMSO): 12.22. ^13^C NMR ([D_6_]DMSO): 54.35 (d, ^2^J(P,*C*) = 4.2 Hz 2 C, CH_3_), 30.22 (d, ^2^J(P,*C*) = 2.7 Hz, 2 C, CH), 27.30 (d, ^3^J(P,*C*) = 4.4 Hz, 4 C, CH_2_), 25.60 (*s*), 25.00 (*s*). ^1^H NMR ([D_6_]DMSO): 1.02 (m, 2 H), 1.17 (m, 4 H), 1.48 (m, 8 H), 1.73 (m, 4 H), 2.49 (s, 6 H), 3.27 (m, 2 H), 10.23 (b, 1 H, NH). IR (KBr, cm^-1^): 3067, 2925, 2802, 1735 (C?O), 1498, 1271, 1236, 1202, 1158, 1005, 980, 893, 851. Raman (cm^-1^): 2929, 2858, 1736, 1446, 1341, 1259, 1188, 1151, 1025, 857, 808, 742, 533, 493, 442, 308. MS (70 ev) m/*z* (%): 383 (20, [*M*]^+^), 368 (2, [M—CH_3_]^+^), 340 (36, [M—C(O)NH]^+^), 271 (35, [P(O)(N(CH_3_)(C_6_H_11_))_2_]^+^), 112 (100, [N(CH_3_)(C_6_H_11_)]^+^), 97 (58, [CF_3_C(O)]^+^), 69 (98, [CF_3_]^+^). Synthesis of [NH_2_CH_3_C_6_H_11_][CF_3_CONPO(NCH_3_C_6_H_11_)_2_] Lithium hydroxide (0.04 g, 1.6 mmol) was added to a solution of CF_3_CONHPO(NCH_3_C_6_H_11_)_2_ (0.62 g, 1.6 mmol) in 10 ml of aqueous methanol (1:3). The solution was stirred at room temperature for 24 h. Colorless single-crystal was obtained after a week at room temperature. Yield: 0.48 g, 60%. Anal. Calc. for C_23_H_44_F_3_N_4_O_2_P: C, 55.59; H, 8.86; N, 11.28. Found: C, 55.47; H, 8.80; N, 11.52%. ^31^P NMR ([D_6_]DMSO): 18.61. ^13^C NMR ([D_6_]DMSO): 23.84 (*s*), 24.83 (*s*), 25.37 (*s*), 25.87 (*s*), 27.20 (d, J(P,*C*)=3.9 Hz), 28.69 (*s*), 29.70 (*s*), 30.38 (*s*), 53.48 (*s*), 56.72 (*s*), 137.58 (dq, CF_3_), 157.14 (q, C?O). IR (KBr, cm^-1^): 3338, 3058, 2936, 2849, 2690, 2624, 1689 (C?O), 1631, 1601, 1553, 1520, 1430, 1375, 1220, 1067, 987, 896, 769, 682.

### Refinement

The hydrogen atoms of NH_2_ group were found in difference Fourier synthesis. The H(C) atom positions were calculated. All hydrogen atoms were refined in isotropic approximation in riding model with with the *U*_iso_(H) parameters equal to 1.2 *U*_eq_(Ci), for methyl groups equal to 1.5 *U*_eq_(Cii), where U(Ci) and U(Cii) are respectively the equivalent thermal parameters of the carbon atoms to which corresponding H atoms are bonded.

### Crystal data

**Table:**

C_7_H_16_N^+^·C_16_H_28_F_3_N_3_O_2_P^−^	*F*(000) = 1072
*M**_r_* = 496.59	*D*_x_ = 1.260 Mg m^−^^3^
Monoclinic, *P*2_1_/*c*	Mo *K*α radiation, λ = 0.71073 Å
Hall symbol: -P 2ybc	Cell parameters from 365 reflections
*a* = 9.183 (3) Å	θ = 2–25°
*b* = 30.893 (7) Å	µ = 0.15 mm^−^^1^
*c* = 9.241 (2) Å	*T* = 120 K
β = 93.039 (7)°	Prism, colorless
*V* = 2617.9 (12) Å^3^	0.40 × 0.30 × 0.25 mm
*Z* = 4	

### Data collection

**Table:**

Bruker SMART 1000 CCD area-detector diffractometer	5148 independent reflections
Radiation source: fine-focus sealed tube	2673 reflections with *I* > 2σ(*I*)
graphite	*R*_int_ = 0.064
φ and ω scans	θ_max_ = 26.0°, θ_min_ = 2.2°
Absorption correction: multi-scan (*SADABS*; Sheldrick, 1996)	*h* = −11→11
*T*_min_ = 0.947, *T*_max_ = 0.969	*k* = −38→37
23153 measured reflections	*l* = −11→11

### Refinement

**Table:**

Refinement on *F*^2^	Primary atom site location: structure-invariant direct methods
Least-squares matrix: full	Secondary atom site location: difference Fourier map
*R*[*F*^2^ > 2σ(*F*^2^)] = 0.060	Hydrogen site location: inferred from neighbouring sites
*wR*(*F*^2^) = 0.113	H-atom parameters constrained
*S* = 1.08	*w* = 1/[σ^2^(*F*_o_^2^) + (0.0126*P*)^2^ + 2.4*P*] where *P* = (*F*_o_^2^ + 2*F*_c_^2^)/3
5148 reflections	(Δ/σ)_max_ = 0.004
304 parameters	Δρ_max_ = 0.33 e Å^−^^3^
0 restraints	Δρ_min_ = −0.32 e Å^−^^3^

### Special details

**Table:**

Geometry. All e.s.d.'s (except the e.s.d. in the dihedral angle between two l.s. planes) are estimated using the full covariance matrix. The cell e.s.d.'s are taken into account individually in the estimation of e.s.d.'s in distances, angles and torsion angles; correlations between e.s.d.'s in cell parameters are only used when they are defined by crystal symmetry. An approximate (isotropic) treatment of cell e.s.d.'s is used for estimating e.s.d.'s involving l.s. planes.
Refinement. Refinement of *F*^2^ against ALL reflections. The weighted *R*-factor *wR* and goodness of fit *S* are based on *F*^2^, conventional *R*-factors *R* are based on *F*, with *F* set to zero for negative *F*^2^. The threshold expression of *F*^2^ > σ(*F*^2^) is used only for calculating *R*-factors(gt) *etc*. and is not relevant to the choice of reflections for refinement. *R*-factors based on *F*^2^ are statistically about twice as large as those based on *F*, and *R*- factors based on ALL data will be even larger.

### Fractional atomic coordinates and isotropic or equivalent isotropic displacement parameters (Å^2^)

**Table:**

	*x*	*y*	*z*	*U*_iso_*/*U*_eq_	
P1	0.59626 (9)	0.11209 (3)	0.00482 (9)	0.0277 (2)	
F1	0.8519 (2)	0.13212 (8)	−0.3599 (2)	0.0674 (7)	
F2	1.0171 (2)	0.15024 (6)	−0.2005 (2)	0.0605 (6)	
F3	1.0165 (2)	0.08688 (6)	−0.2932 (2)	0.0498 (5)	
O1	0.5406 (2)	0.06625 (6)	0.0163 (2)	0.0306 (5)	
O2	0.9035 (2)	0.07648 (7)	−0.0331 (2)	0.0381 (6)	
N1	0.7111 (3)	0.12011 (8)	−0.1208 (3)	0.0285 (6)	
N2	0.4692 (3)	0.14801 (7)	−0.0417 (3)	0.0250 (6)	
N3	0.6583 (3)	0.12719 (7)	0.1670 (3)	0.0280 (6)	
N4	0.3080 (3)	0.01010 (8)	0.0416 (3)	0.0300 (6)	
H4NA	0.3810	0.0319	0.0427	0.029 (9)*	
H4NB	0.3547	−0.0172	0.0350	0.051 (11)*	
C1	0.8390 (3)	0.10240 (10)	−0.1197 (3)	0.0302 (8)	
C2	0.9298 (3)	0.11769 (11)	−0.2449 (4)	0.0330 (8)	
C3	0.3392 (3)	0.14748 (10)	0.0448 (3)	0.0348 (8)	
H3A	0.2959	0.1765	0.0455	0.052*	
H3B	0.3672	0.1387	0.1444	0.052*	
H3C	0.2679	0.1269	0.0023	0.052*	
C4	0.4473 (3)	0.16366 (9)	−0.1935 (3)	0.0259 (7)	
H4A	0.5466	0.1700	−0.2277	0.031*	
C5	0.3796 (3)	0.13003 (10)	−0.2974 (3)	0.0317 (8)	
H5A	0.2808	0.1225	−0.2674	0.038*	
H5B	0.4395	0.1034	−0.2929	0.038*	
C6	0.3694 (4)	0.14714 (11)	−0.4532 (3)	0.0411 (9)	
H6A	0.4689	0.1512	−0.4873	0.049*	
H6B	0.3186	0.1256	−0.5170	0.049*	
C7	0.2875 (4)	0.18992 (11)	−0.4631 (4)	0.0443 (9)	
H7A	0.2889	0.2012	−0.5633	0.053*	
H7B	0.1845	0.1851	−0.4406	0.053*	
C8	0.3553 (4)	0.22305 (10)	−0.3587 (4)	0.0411 (9)	
H8A	0.2965	0.2499	−0.3638	0.049*	
H8B	0.4547	0.2302	−0.3877	0.049*	
C9	0.3634 (4)	0.20606 (10)	−0.2029 (3)	0.0340 (8)	
H9A	0.4125	0.2277	−0.1381	0.041*	
H9B	0.2636	0.2015	−0.1703	0.041*	
C10	0.6862 (4)	0.09517 (10)	0.2826 (3)	0.0391 (9)	
H10A	0.6866	0.1096	0.3770	0.059*	
H10B	0.7811	0.0815	0.2709	0.059*	
H10C	0.6095	0.0731	0.2769	0.059*	
C11	0.7380 (3)	0.16853 (9)	0.1839 (3)	0.0292 (8)	
H11A	0.7251	0.1840	0.0889	0.035*	
C12	0.9025 (3)	0.16295 (10)	0.2143 (4)	0.0357 (8)	
H12A	0.9204	0.1477	0.3079	0.043*	
H12B	0.9424	0.1449	0.1374	0.043*	
C13	0.9808 (4)	0.20648 (11)	0.2202 (4)	0.0472 (10)	
H13A	1.0859	0.2019	0.2448	0.057*	
H13B	0.9710	0.2205	0.1239	0.057*	
C14	0.9170 (4)	0.23579 (11)	0.3327 (4)	0.0504 (10)	
H14A	0.9658	0.2643	0.3314	0.060*	
H14B	0.9357	0.2230	0.4302	0.060*	
C15	0.7527 (4)	0.24187 (11)	0.3035 (4)	0.0482 (10)	
H15A	0.7342	0.2574	0.2106	0.058*	
H15B	0.7133	0.2597	0.3815	0.058*	
C16	0.6749 (4)	0.19793 (10)	0.2965 (4)	0.0403 (9)	
H16A	0.6849	0.1838	0.3927	0.048*	
H16B	0.5697	0.2024	0.2722	0.048*	
C17	0.2070 (3)	0.01882 (10)	−0.0858 (3)	0.0367 (9)	
H17A	0.2635	0.0236	−0.1715	0.055*	
H17B	0.1490	0.0447	−0.0674	0.055*	
H17C	0.1420	−0.0060	−0.1026	0.055*	
C18	0.2394 (3)	0.00821 (10)	0.1845 (3)	0.0299 (8)	
H18A	0.1527	−0.0114	0.1745	0.036*	
C19	0.3478 (4)	−0.01118 (10)	0.2964 (3)	0.0364 (9)	
H19A	0.4363	0.0071	0.3050	0.044*	
H19B	0.3770	−0.0404	0.2646	0.044*	
C20	0.2801 (4)	−0.01433 (11)	0.4438 (3)	0.0430 (9)	
H20A	0.1985	−0.0353	0.4375	0.052*	
H20B	0.3541	−0.0252	0.5167	0.052*	
C21	0.2246 (4)	0.02903 (11)	0.4918 (4)	0.0454 (10)	
H21A	0.3080	0.0489	0.5107	0.054*	
H21B	0.1745	0.0254	0.5833	0.054*	
C22	0.1197 (4)	0.04878 (11)	0.3780 (3)	0.0437 (9)	
H22A	0.0902	0.0779	0.4102	0.052*	
H22B	0.0309	0.0306	0.3674	0.052*	
C23	0.1878 (4)	0.05249 (10)	0.2320 (3)	0.0357 (8)	
H23A	0.1151	0.0641	0.1590	0.043*	
H23B	0.2714	0.0728	0.2397	0.043*	

### Atomic displacement parameters (Å^2^)

**Table:**

	*U*^11^	*U*^22^	*U*^33^	*U*^12^	*U*^13^	*U*^23^
P1	0.0272 (5)	0.0257 (5)	0.0301 (5)	−0.0010 (4)	0.0017 (4)	0.0008 (4)
F1	0.0449 (13)	0.112 (2)	0.0460 (13)	0.0198 (13)	0.0116 (11)	0.0314 (13)
F2	0.0560 (14)	0.0487 (13)	0.0793 (16)	−0.0186 (11)	0.0286 (12)	−0.0111 (12)
F3	0.0466 (13)	0.0509 (13)	0.0535 (13)	0.0069 (10)	0.0170 (10)	−0.0057 (11)
O1	0.0307 (12)	0.0241 (12)	0.0372 (13)	−0.0017 (10)	0.0042 (10)	0.0018 (10)
O2	0.0367 (14)	0.0360 (14)	0.0416 (14)	0.0078 (11)	0.0014 (11)	0.0029 (11)
N1	0.0212 (14)	0.0337 (16)	0.0304 (15)	0.0044 (12)	0.0008 (12)	0.0002 (12)
N2	0.0265 (15)	0.0242 (14)	0.0249 (14)	−0.0005 (11)	0.0056 (12)	0.0033 (11)
N3	0.0345 (16)	0.0223 (14)	0.0268 (15)	−0.0040 (12)	−0.0019 (12)	0.0018 (12)
N4	0.0331 (16)	0.0260 (16)	0.0308 (16)	0.0019 (13)	0.0009 (13)	0.0012 (12)
C1	0.031 (2)	0.0290 (19)	0.0299 (19)	−0.0028 (16)	−0.0004 (16)	−0.0024 (15)
C2	0.0264 (19)	0.032 (2)	0.040 (2)	0.0042 (16)	−0.0021 (16)	−0.0007 (16)
C3	0.036 (2)	0.0320 (19)	0.037 (2)	0.0024 (16)	0.0092 (17)	0.0039 (16)
C4	0.0234 (17)	0.0253 (17)	0.0294 (18)	0.0005 (14)	0.0044 (14)	0.0060 (14)
C5	0.0325 (19)	0.0283 (18)	0.0338 (19)	0.0005 (15)	−0.0027 (15)	0.0034 (15)
C6	0.050 (2)	0.039 (2)	0.034 (2)	−0.0105 (18)	−0.0064 (17)	−0.0030 (17)
C7	0.040 (2)	0.048 (2)	0.044 (2)	−0.0035 (18)	−0.0075 (18)	0.0165 (19)
C8	0.044 (2)	0.032 (2)	0.047 (2)	0.0049 (17)	−0.0007 (18)	0.0093 (17)
C9	0.037 (2)	0.0297 (19)	0.036 (2)	−0.0004 (15)	0.0036 (16)	0.0043 (16)
C10	0.054 (2)	0.0310 (19)	0.031 (2)	−0.0043 (17)	−0.0053 (17)	0.0073 (16)
C11	0.035 (2)	0.0261 (18)	0.0258 (18)	−0.0052 (15)	−0.0029 (15)	0.0027 (14)
C12	0.036 (2)	0.038 (2)	0.033 (2)	−0.0044 (16)	0.0010 (16)	−0.0048 (16)
C13	0.043 (2)	0.053 (2)	0.045 (2)	−0.0163 (19)	−0.0068 (19)	0.0032 (19)
C14	0.060 (3)	0.035 (2)	0.054 (3)	−0.0102 (19)	−0.015 (2)	−0.0093 (19)
C15	0.053 (3)	0.036 (2)	0.055 (3)	0.0040 (18)	−0.005 (2)	−0.0125 (19)
C16	0.040 (2)	0.033 (2)	0.047 (2)	0.0020 (17)	−0.0012 (18)	−0.0059 (17)
C17	0.041 (2)	0.041 (2)	0.0278 (19)	0.0017 (17)	−0.0061 (16)	0.0043 (16)
C18	0.032 (2)	0.0329 (19)	0.0247 (18)	−0.0010 (15)	0.0033 (15)	0.0027 (15)
C19	0.041 (2)	0.031 (2)	0.036 (2)	0.0006 (16)	−0.0051 (17)	0.0008 (16)
C20	0.058 (3)	0.038 (2)	0.032 (2)	−0.0026 (18)	−0.0092 (18)	0.0079 (17)
C21	0.062 (3)	0.047 (2)	0.027 (2)	−0.001 (2)	0.0042 (18)	−0.0015 (17)
C22	0.051 (2)	0.045 (2)	0.036 (2)	0.0031 (18)	0.0105 (18)	−0.0010 (18)
C23	0.042 (2)	0.034 (2)	0.031 (2)	0.0049 (16)	−0.0007 (16)	0.0026 (15)

### Geometric parameters (Å, °)

**Table:**

P1—O1	1.511 (2)	C10—H10B	0.9800
P1—N1	1.629 (3)	C10—H10C	0.9800
P1—N3	1.643 (2)	C11—C16	1.519 (4)
P1—N2	1.651 (2)	C11—C12	1.531 (4)
F1—C2	1.326 (3)	C11—H11A	1.0000
F2—C2	1.337 (3)	C12—C13	1.525 (4)
F3—C2	1.333 (3)	C12—H12A	0.9900
O2—C1	1.258 (3)	C12—H12B	0.9900
N1—C1	1.295 (4)	C13—C14	1.520 (5)
N2—C3	1.472 (3)	C13—H13A	0.9900
N2—C4	1.487 (3)	C13—H13B	0.9900
N3—C10	1.468 (4)	C14—C15	1.530 (5)
N3—C11	1.476 (3)	C14—H14A	0.9900
N4—C17	1.484 (4)	C14—H14B	0.9900
N4—C18	1.494 (4)	C15—C16	1.534 (4)
N4—H4NA	0.9502	C15—H15A	0.9900
N4—H4NB	0.9499	C15—H15B	0.9900
C1—C2	1.536 (4)	C16—H16A	0.9900
C3—H3A	0.9800	C16—H16B	0.9900
C3—H3B	0.9800	C17—H17A	0.9800
C3—H3C	0.9800	C17—H17B	0.9800
C4—C9	1.520 (4)	C17—H17C	0.9800
C4—C5	1.525 (4)	C18—C23	1.520 (4)
C4—H4A	1.0000	C18—C19	1.520 (4)
C5—C6	1.531 (4)	C18—H18A	1.0000
C5—H5A	0.9900	C19—C20	1.530 (4)
C5—H5B	0.9900	C19—H19A	0.9900
C6—C7	1.521 (4)	C19—H19B	0.9900
C6—H6A	0.9900	C20—C21	1.509 (4)
C6—H6B	0.9900	C20—H20A	0.9900
C7—C8	1.517 (4)	C20—H20B	0.9900
C7—H7A	0.9900	C21—C22	1.516 (4)
C7—H7B	0.9900	C21—H21A	0.9900
C8—C9	1.531 (4)	C21—H21B	0.9900
C8—H8A	0.9900	C22—C23	1.521 (4)
C8—H8B	0.9900	C22—H22A	0.9900
C9—H9A	0.9900	C22—H22B	0.9900
C9—H9B	0.9900	C23—H23A	0.9900
C10—H10A	0.9800	C23—H23B	0.9900
			
O1—P1—N1	115.26 (13)	N3—C11—C12	113.6 (2)
O1—P1—N3	107.72 (12)	C16—C11—C12	110.5 (3)
N1—P1—N3	113.65 (13)	N3—C11—H11A	106.5
O1—P1—N2	114.29 (12)	C16—C11—H11A	106.5
N1—P1—N2	100.79 (12)	C12—C11—H11A	106.5
N3—P1—N2	104.66 (12)	C13—C12—C11	111.5 (3)
C1—N1—P1	123.4 (2)	C13—C12—H12A	109.3
C3—N2—C4	116.3 (2)	C11—C12—H12A	109.3
C3—N2—P1	115.67 (19)	C13—C12—H12B	109.3
C4—N2—P1	121.53 (19)	C11—C12—H12B	109.3
C10—N3—C11	116.1 (2)	H12A—C12—H12B	108.0
C10—N3—P1	120.7 (2)	C14—C13—C12	110.6 (3)
C11—N3—P1	119.19 (19)	C14—C13—H13A	109.5
C17—N4—C18	115.7 (2)	C12—C13—H13A	109.5
C17—N4—H4NA	106.9	C14—C13—H13B	109.5
C18—N4—H4NA	110.4	C12—C13—H13B	109.5
C17—N4—H4NB	112.1	H13A—C13—H13B	108.1
C18—N4—H4NB	103.6	C13—C14—C15	111.4 (3)
H4NA—N4—H4NB	108.1	C13—C14—H14A	109.3
O2—C1—N1	132.1 (3)	C15—C14—H14A	109.3
O2—C1—C2	114.8 (3)	C13—C14—H14B	109.3
N1—C1—C2	113.1 (3)	C15—C14—H14B	109.3
F1—C2—F3	106.1 (3)	H14A—C14—H14B	108.0
F1—C2—F2	106.4 (3)	C14—C15—C16	110.6 (3)
F3—C2—F2	106.3 (3)	C14—C15—H15A	109.5
F1—C2—C1	114.6 (3)	C16—C15—H15A	109.5
F3—C2—C1	113.0 (3)	C14—C15—H15B	109.5
F2—C2—C1	109.9 (3)	C16—C15—H15B	109.5
N2—C3—H3A	109.5	H15A—C15—H15B	108.1
N2—C3—H3B	109.5	C11—C16—C15	111.4 (3)
H3A—C3—H3B	109.5	C11—C16—H16A	109.4
N2—C3—H3C	109.5	C15—C16—H16A	109.4
H3A—C3—H3C	109.5	C11—C16—H16B	109.4
H3B—C3—H3C	109.5	C15—C16—H16B	109.4
N2—C4—C9	112.1 (2)	H16A—C16—H16B	108.0
N2—C4—C5	113.8 (2)	N4—C17—H17A	109.5
C9—C4—C5	111.3 (2)	N4—C17—H17B	109.5
N2—C4—H4A	106.4	H17A—C17—H17B	109.5
C9—C4—H4A	106.4	N4—C17—H17C	109.5
C5—C4—H4A	106.4	H17A—C17—H17C	109.5
C4—C5—C6	111.1 (2)	H17B—C17—H17C	109.5
C4—C5—H5A	109.4	N4—C18—C23	111.9 (2)
C6—C5—H5A	109.4	N4—C18—C19	108.9 (2)
C4—C5—H5B	109.4	C23—C18—C19	111.2 (3)
C6—C5—H5B	109.4	N4—C18—H18A	108.2
H5A—C5—H5B	108.0	C23—C18—H18A	108.2
C7—C6—C5	111.2 (3)	C19—C18—H18A	108.2
C7—C6—H6A	109.4	C18—C19—C20	110.4 (3)
C5—C6—H6A	109.4	C18—C19—H19A	109.6
C7—C6—H6B	109.4	C20—C19—H19A	109.6
C5—C6—H6B	109.4	C18—C19—H19B	109.6
H6A—C6—H6B	108.0	C20—C19—H19B	109.6
C8—C7—C6	111.3 (3)	H19A—C19—H19B	108.1
C8—C7—H7A	109.4	C21—C20—C19	111.4 (3)
C6—C7—H7A	109.4	C21—C20—H20A	109.3
C8—C7—H7B	109.4	C19—C20—H20A	109.3
C6—C7—H7B	109.4	C21—C20—H20B	109.3
H7A—C7—H7B	108.0	C19—C20—H20B	109.3
C7—C8—C9	111.4 (3)	H20A—C20—H20B	108.0
C7—C8—H8A	109.3	C20—C21—C22	111.5 (3)
C9—C8—H8A	109.3	C20—C21—H21A	109.3
C7—C8—H8B	109.3	C22—C21—H21A	109.3
C9—C8—H8B	109.3	C20—C21—H21B	109.3
H8A—C8—H8B	108.0	C22—C21—H21B	109.3
C4—C9—C8	110.4 (3)	H21A—C21—H21B	108.0
C4—C9—H9A	109.6	C21—C22—C23	111.7 (3)
C8—C9—H9A	109.6	C21—C22—H22A	109.3
C4—C9—H9B	109.6	C23—C22—H22A	109.3
C8—C9—H9B	109.6	C21—C22—H22B	109.3
H9A—C9—H9B	108.1	C23—C22—H22B	109.3
N3—C10—H10A	109.5	H22A—C22—H22B	107.9
N3—C10—H10B	109.5	C18—C23—C22	109.7 (3)
H10A—C10—H10B	109.5	C18—C23—H23A	109.7
N3—C10—H10C	109.5	C22—C23—H23A	109.7
H10A—C10—H10C	109.5	C18—C23—H23B	109.7
H10B—C10—H10C	109.5	C22—C23—H23B	109.7
N3—C11—C16	112.6 (3)	H23A—C23—H23B	108.2
			
O1—P1—N1—C1	−64.8 (3)	C4—C5—C6—C7	−54.9 (4)
N3—P1—N1—C1	60.3 (3)	C5—C6—C7—C8	55.0 (4)
N2—P1—N1—C1	171.7 (2)	C6—C7—C8—C9	−55.9 (4)
O1—P1—N2—C3	51.3 (2)	N2—C4—C9—C8	174.9 (2)
N1—P1—N2—C3	175.5 (2)	C5—C4—C9—C8	−56.4 (3)
N3—P1—N2—C3	−66.3 (2)	C7—C8—C9—C4	56.4 (4)
O1—P1—N2—C4	−99.4 (2)	C10—N3—C11—C16	−75.6 (3)
N1—P1—N2—C4	24.9 (2)	P1—N3—C11—C16	126.7 (2)
N3—P1—N2—C4	143.0 (2)	C10—N3—C11—C12	51.0 (4)
O1—P1—N3—C10	14.9 (3)	P1—N3—C11—C12	−106.8 (3)
N1—P1—N3—C10	−114.1 (2)	N3—C11—C12—C13	176.0 (3)
N2—P1—N3—C10	136.9 (2)	C16—C11—C12—C13	−56.3 (4)
O1—P1—N3—C11	171.6 (2)	C11—C12—C13—C14	56.4 (4)
N1—P1—N3—C11	42.6 (3)	C12—C13—C14—C15	−56.2 (4)
N2—P1—N3—C11	−66.4 (2)	C13—C14—C15—C16	55.9 (4)
P1—N1—C1—O2	1.1 (5)	N3—C11—C16—C15	−175.8 (3)
P1—N1—C1—C2	−176.0 (2)	C12—C11—C16—C15	56.0 (4)
O2—C1—C2—F1	157.9 (3)	C14—C15—C16—C11	−55.9 (4)
N1—C1—C2—F1	−24.4 (4)	C17—N4—C18—C23	−69.9 (3)
O2—C1—C2—F3	36.2 (4)	C17—N4—C18—C19	166.7 (3)
N1—C1—C2—F3	−146.1 (3)	N4—C18—C19—C20	−179.0 (3)
O2—C1—C2—F2	−82.3 (3)	C23—C18—C19—C20	57.2 (3)
N1—C1—C2—F2	95.3 (3)	C18—C19—C20—C21	−55.3 (4)
C3—N2—C4—C9	48.6 (3)	C19—C20—C21—C22	54.5 (4)
P1—N2—C4—C9	−161.0 (2)	C20—C21—C22—C23	−55.5 (4)
C3—N2—C4—C5	−78.8 (3)	N4—C18—C23—C22	−179.7 (3)
P1—N2—C4—C5	71.7 (3)	C19—C18—C23—C22	−57.7 (4)
N2—C4—C5—C6	−176.2 (2)	C21—C22—C23—C18	56.5 (4)
C9—C4—C5—C6	56.0 (3)		

### Hydrogen-bond geometry (Å, °)

**Table:**

*D*—H···*A*	*D*—H	H···*A*	*D*···*A*	*D*—H···*A*
N4—H4NA···O1	0.95	1.84	2.771 (3)	167
N4—H4NB···O1^i^	0.95	1.87	2.804 (3)	168

Symmetry codes:  (i) −*x*+1, −*y*, −*z*.
